# pERK-mediated IL8 secretion can enhance the migration, invasion, and cisplatin resistance of CD10-positive oral cancer cells

**DOI:** 10.1186/s12885-021-09025-7

**Published:** 2021-12-01

**Authors:** Yinfei Pu, Qingxiang Li, Yifei Wang, Le Xu, Qiao Qiao, Yuxing Guo, Chuanbin Guo

**Affiliations:** 1 Department of Oral and Maxillofacial Surgery, Peking University School and Hospital of Stomatology, NO. 22, Zhongguancun South Street, Haidian District, Beijing, 100081 China; 2 Second Clinical Division, Peking University School and Hospital of Stomatology, Beijing, 100081 P. R. China; 3 National Clinical Research Center for Oral Diseases, NO. 22, Zhongguancun South Street, Haidian District, Beijing, 100081 China; 4 National Engineering Laboratory for Digital and Material Technology of Stomatology, NO. 22, Zhongguancun South Street, Haidian District, Beijing, 100081 China; 5 Beijing Key Laboratory of Digital Stomatology, Peking University School and Hospital of Stomatology, NO. 22, Zhongguancun South Street, Haidian District, Beijing, 100081 China

**Keywords:** IL8, CD10, p-ERK, Oral squamous cell carcinoma, Drug resistance, Cisplatin

## Abstract

**Background:**

Cancer stem cells (CSCs) drive tumor initiation and progression and participate in tumor chemoresistance. We recently discovered that oral squamous cell carcinoma (OSCC) cells that highly express CD10 (CD10H cells) present cancer stem cells (CSC)-associated characteristics, which, in turn, affect the tumor growth, epithelial-mesenchymal transition (EMT), and resistance to cisplatin. In this study, we further investigated this mechanism *in vitro* and *in vivo*. We hypothesized that IL8 might regulate migration, invasion, and cisplatin resistance of CD10-positive oral cancer cells through the ERK pathway.

**Methods:**

CD10 MicroBead Kit was used to select HN6 cells with high and low expression of CD10. The target protein IL8 was screened via protein chip assay. Lentiviral transduction and specific inhibitor were applied to investigate the signaling pathway. Real-time PCR, Western blot, and immunohistochemistry were used to analyze the mRNA and protein expression; transwell assay, spheroid formation assay, and cell viability assay were used to study the cell biological behavior *in vitro*; xenograft animal model was used to evaluate the tumor formation rate *in vivo*.

**Results:**

Overexpression of CD10 promoted CSC-related genes expression and enhanced migration, invasion, spheroid formation, and chemoresistance in HN6 cells. Moreover, the overexpression of IL8 was detected in OSCC tumor tissue and cell lines (HN6 and CAL27) overexpressing CD10. IL8 secreted by CD10H HN6 promoted migration and invasion and restored tumor chemosensitivity via the p-ERK signaling pathway, while the inhibition of IL8 increased the chemosensitivity to cisplatin.

**Conclusions:**

IL8 secretion by CD10 positive cells promotes migration, invasion, and cisplatin resistance of OSCC via the p-ERK signaling pathway.

**Supplementary Information:**

The online version contains supplementary material available at 10.1186/s12885-021-09025-7.

## Background

Oral cancer is a type of cancer that affects the lips, tongue, floor of the month, cheeks, sinuses, hard and soft palate, and throat. Despite improvements in early diagnosis and treatment, the 5-year survival rate of oral cancer is still very low, ranging from 40.2 to 50.6% [1]. Surgery is the standard treatment for primary oral cancer, while radiation therapy and chemotherapy are commonly used as adjuvant treatments [2]. Local recurrence, regional lymphatic metastasis, and chemo-radiotherapy resistance are common causes associated with treatment failure in patients with oral squamous cell carcinoma (OSCC) [3].

Self-renewing cancer stem cells (CSCs) are a subpopulation of stem cells that drive tumor initiation and progression [4]. Compared to other tumor cells, CSCs are more resistant to chemotherapy [5] and radiotherapy [6, 7] and have more aggressive biological behavior [8]. Biomarkers expressed on the surface of CSCs provide targeted molecular therapies for various cancers [9]. Yet, considering the functional heterogeneity and hierarchical organization of CSCs, surface markers may change.

CD44 and CD133 are two of the most widely studied stem cells' surface markers in oral cancer [10]. A recent study suggested that a high level of CD10 expression has been associated with poor overall survival and cisplatin resistance in patients with head and neck cancer [11]. Moreover, we discovered that OSCC cells that highly express CD10 (CD10H cells) present CSC-associated characteristics, which, in turn, affect the tumor growth, EMT, and resistance to cisplatin [12]. Yet, the exact mechanism leading to this behavior remains unclear. In this study, we further investigated the mechanism through which CD10H oral cancer cells regulate their behavior (migration, invasion, and cisplatin resistance).

IL8, also known as CXCL8, is a pro-inflammatory chemokine from the CXC family [13]. Its high expression is related to tumor formation and progression in many types of cancer [14–16]. IL8 promotes liver cancer migration [17] and accelerates breast cancer [18] and gastric cancer [19] migration and invasion [18]. Moreover, IL8 increases the survival of cancer stem-like cell populations in breast cancer [20]. Peng *et al* found that IL8 confer higher cancer stemness and leads to chemoresistance [21]. Tamatani and colleagues also demonstrated that IL8 regulates chemoresistance in oral cancer [22].

IL8 regulates various signaling pathways, such as ERK [23, 24], PI3K-AKT [25], and p38 MAPK [26]. ERK pathway can regulate the expression of IL8 in triple-negative breast cancer, thereby affecting the characteristics of breast cancer metastasis [27]. Moreover, IL8 participates in EMT and regulates cancer stem cells (CSC) through ERK [23, 24]. In this study, we hypothesized that IL8 might regulate the biological behavior of CD10-positive oral cancer cells through the ERK pathway.

## Methods

### Cell culture

The human oral squamous cell carcinoma cell lines CAL27 (RRID: CVCL_1107) and HN6 (RRID: CVCL_5516) were used in this study. HN6 was obtained from the Central Laboratory of Peking University School and Hospital of Stomatology, and the CAL27 was obtained from the American Type Culture Collection (ATCC, CRL-2095). The cells were cultured in DMEM (Life Technology) containing 1% penicillin/streptomycin (Gibco) and 10% FBS (Gibco) in a humidified atmosphere containing 5% CO_2_/95% air at 37°C. The cells were verified by STR analysis and mycoplasma detection. All cells were cryopreserved for more than 6 months, and the general length of time between thawing and use did not exceed 3 months.

### Magnetic-activated cell sorting (MACS)

MACS kit (Miltenyi) was used to sort out cells with high expression of CD10 (CD10H) and cells with low expression of CD10 (CD10L). Briefly, 10^7^ cells were resuspended in a 40 μl buffer mixed with 10μl of Anti-CD10-biotin at 4°C for 10 min. Consequently, cells were incubated with 30 μl buffer and 20 μl of anti-biotin-microbeads at 4°C for 15 min. After being washed with the buffer, the cells were flown into the LS and LD sorting columns in order, which were placed in a magnetic field. The unlabeled cells (CD10 low cells) were collected. The LS sorting column was then pulled out from the magnetic field, and the labeled cells (CD10 high cells) were collected.

### Real-time PCR

TRIzol reagent (Ambion) was used for total RNA extraction Ambion) according to the manufacturer's protocol. The spectrophotometer (Bio Tek) was used to analyze the RNA quantity and purity. The cDNA was synthesized using a reverse transcription kit (Promega). Real-time PCR assays were performed in the ABI 7500 Real-Time PCR Detection System (Applied Biosystems) using SYBR Green (Roche, Switzerland). GAPDH was used as the endogenous standard. The PCR program consisted of the following steps: pre-denaturation at 95°C for 10 min; 40 cycles amplification of 95°C for 15 s; 60°C for 1 min. All primers were purchased from Shanghai Shenggong Co., Ltd; the sequences of the primers are shown in Table S1. The relative expression level was normalized to the amount of GAPDH and calculated using the 2^−ΔΔCt^ method.

### Transwell assay

Chemotaxis was measured using a transwell assay (PIEP 12R 48; Millipore; USA). In the mono-culture transwell assay, 1 × 10^5^ tumor cells (200 μL) were planted into the upper compartment with or without Matrigel (BD, USA), while a 1.3 mL complete medium was added to the lower compartment. After 6-12 h, the upper compartment was washed with PBS 3 times, and the cells were fixed with 10% paraformaldehyde for 10 min. The cells were then stained with crystal violet and photographed at 10× magnification (Nikon, Japan) after being wiped from the upper surface of the upper compartment.

### Cell viability assay

The tumor cells were seeded at a density of 5,000 per well in a 96-well culture plate. Cells were then exposed to 10μM of cisplatin (diluted with PBS; Jiangsu Haosen Pharmaceutical Co., Ltd.) for 12, 24, 48h. At the time point, 10 μl of sterile CCK-8 solution (Bimake) was added to each well and incubated for another 1h at 37°C. The absorbance was measured at 450 nm using a microplate reader.

### ELISA

All the cells and supernatants were collected for ELISA. RIPA buffer (Applygen, China) was used to extract total protein. The protein concentration was determined using the bicinchoninic acid reagent (Thermo Fisher Scientific, USA). ELISA was then used to assess the levels of CXCL8 (ab174442; Abcam; USA) in the cytoplasm and supernatant according to the manufacturers’ instructions.

### Western blot

Tumor cells were cultured in 60 mm dishes. RIPA buffer (Applygen, China) was used to extract the total protein. The protein concentration was determined using the bicinchoninic acid reagent (Thermo Fisher Scientific, USA). Samples were then loaded on 10% SDS-PAGE and transferred onto a PVDF (polyvinyl difluoride) membrane. Membranes containing different proteins were immunoprobed with the corresponding antibodies: IL8 (ab18672; Abcam; USA, 1:1000), CD10 (10805-T56; Sina Biological; China, 1:1000); p-ERK (4370T; CST; USA, 1:1000); t-ERK (4695T; CST; USA, 1:1000) overnight at 4°C. Samples were then washed and incubated with Anti-rabbit secondary antibody (7074S; CST; USA) and anti-mouse secondary antibody (7076S; CST; USA)(1:5,000) at room temperature for 2h. The protein concentration was detected using a chemiluminescence detection system (CW0048M; CWBIO; China) and analyzed using Image J software (National Institutes of Health).

### Cell immunohistochemistry

Tumor cells were fixed in 4% paraformaldehyde for 15 min at RT, followed by incubation in 0.1% (v/v) Triton-100-PBS. Subsequently, cells were blocked with 10% goat serum (Zhongshan Biosciences Inc.) and then incubated with CD10 antibody (1:200, Novus) at 4°C overnight. The cells were then incubated with a secondary antibody (Zhongshan Biosciences Inc.) for 1 hr at RT. Nuclear staining was performed by incubation with hematoxylin. The images were then captured using an optimal fluorescent microscope (Olympus).

### Lentiviral Transduction

Lentiviral transduction was used to overexpress IL8 in HN6 (Genechem, Shanghai, China). HN6 were cultured in 1 × 106 TU/mL virus for 12 h, after which the medium was replaced with a fresh one. After culturing for 48 h, cultures with transduction efficiencies >90% were used in analyses.

### Animals

A total of 45 4-week-old female BALB/c-nude mice (Vital River Laboratory Animal Technology) were housed in an environment with a relative humidity of 50 ± 1%, a temperature of 22 ± 1°C, and a light/dark cycle of 12/12 hr. Mat and feed were changed every 2 days. The mice were anesthetized and sacrificed when their weight decreased by 20%. All animal studies were done according to the National Institute of Health (NIH) USA guidelines on the care and use of animals for experimental procedures and according to local laws and regulations. Peking University institutional animal care approved the study, which was conducted according to the AAALAC and the IACUC guidelines (2018/06/27, NO. LA2018249). The study was carried out in compliance with the ARRIVE guidelines.

After 2 weeks, 45 nude mice were randomly divided into 3 groups: HN6 Oe and its controlled group HN6 and HN6 Mock. Every group contained 5 cell concentration including 1 X 10^4^, 5 X 10^4^, 10 X 10^4^, 50 X 10^4^, 100 X 10^4^ cells per site. Three mice and 6 injection sites were designed for every cell concentration to ensure statistical analysis. Mice were anesthetized using 1% pentobarbital sodium (Sigma). Next, tumor cells were subcutaneously injected into the back. Tumor size was measured on days 7, 9, 11, and 13 post cell inoculation. During the experiment, the weight of all mice did not decrease. On day 14, mice were anesthetized and sacrificed with an overdose of 2% pentobarbital sodium (0.5mL).

### Statistical Analysis

Data are represented as the mean ± standard deviation. Statistically significant differences (*p* < 0.05) between the groups were evaluated using one-way analysis of variance or the Student’s t-test. All the statistical analyses were performed using SPSS 19.0 software (IBM Corp., Armonk, NY, USA).

## Results

### Overexpression of CD10 enhanced the migration, invasion, spheroid formation, and chemoresistance in HN6 cells

**Fig. 1:**
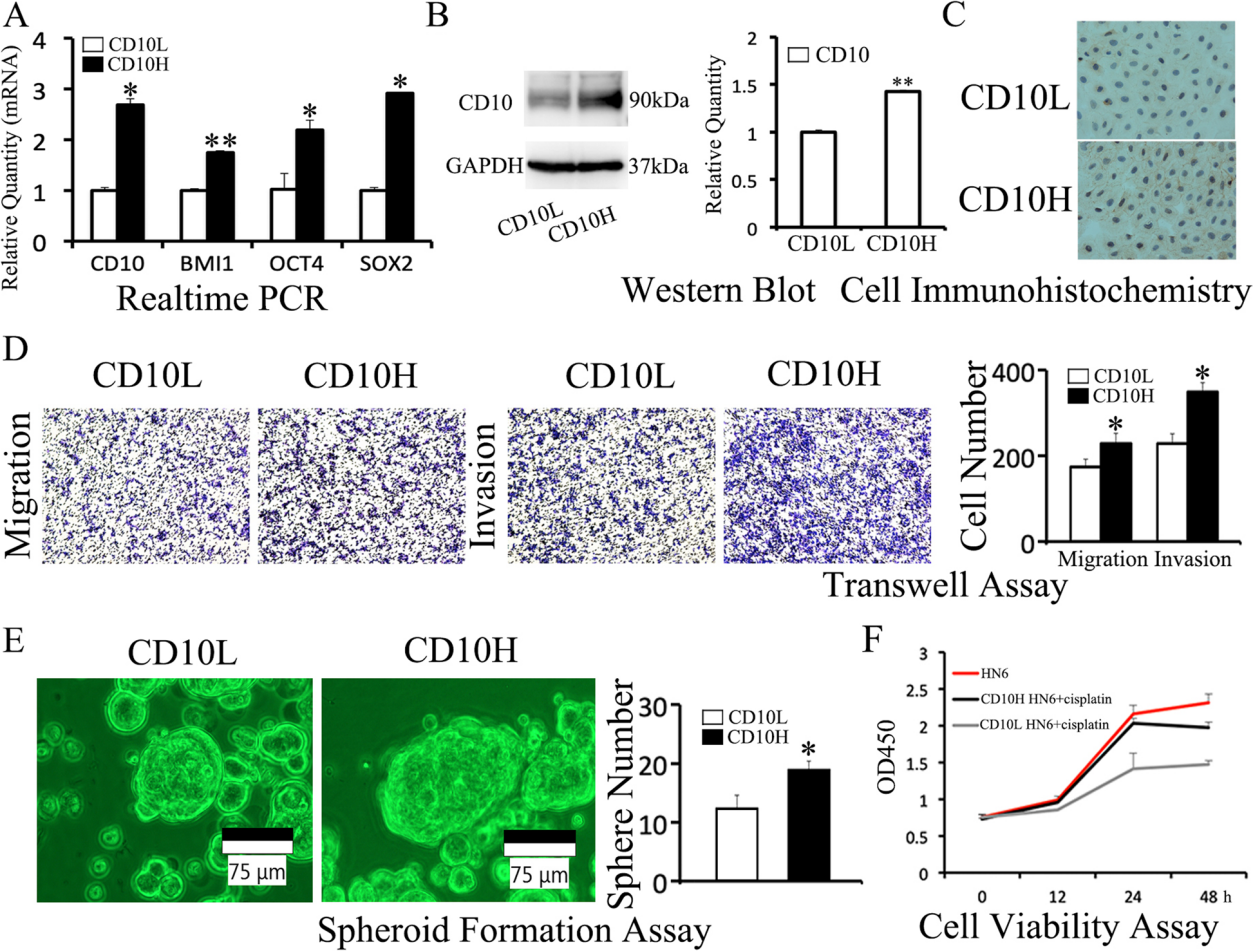
Overexpression of CD10 enhanced the migration, invasion, spheroid formation, and chemoresistance in HN6 cells**.** CD10H HN6 and CD10L HN6 cells were screened by MicroBead Kit and identified by real-time PCR **(A)**, Western blot **(B)**, and cell immonohistochemistry **(C)**. The migration and invasion ability was evaluated by transwell assay **(D)**. The spheroid formation ability was evaluated by spheroid formation assay **(E)**. Cell viability after cisplatin treatment in HN6 cell lines was detected by CCK-8 assay **(F)**. *P<0.05, **P<0.01

CD10 MicroBead Kit was used to screen CD10H expression HN6 cells. The results showed that the mRNA level of CD10 was 2.69±0.12 in CD10H HN6 compared to 1±0.06 in CD10L HN6 (*P*<0.05, Fig. 1A). In addition, CSC-related BMI1 (1±0.03 v.s. 1.74±0.04), OCT4 (1±0.31 v.s. 2.2±0.19), and SOX2 (1±0.06 v.s. 2.91±0.01) were up-regulated in CD10H HN6 (Fig. 1A). Furthermore, Western blot (1.43±0.02 v.s. 1±0.02, Fig. 1B) and cell immunohistochemistry (Fig. 1C) showed a high expression of CD10 protein in CD10H HN6 cells compared to CD10L HN6 cells.

Next, we evaluated the effect of CD10 on cell biological behavior (migration, invasion, spheroid formation, and chemoresistance). The number of migrating cells (229±24 v.s. 175±18, Fig. 1D) and invading cells (350±22 v.s. 229±23, Fig. 1D) was higher in the CD10H HN6 group compared to the CD10L HN6 group. In addition, spheroidal cell aggregates (19±1.4 v.s. 12±1.4, Fig. 1E) were higher in the CD10H HN6 group than in the CD10L HN6 group. Moreover, cell viability was significantly higher in the CD10H HN6 group compared to the CD10L group when tumor cells were treated with cisplatin for 24 and 48h (Fig. 1F). This data suggested that the overexpression of CD10 enhances the migration, invasion, spheroid formation, and chemoresistance in HN6 cells.

### IL8 expression was higher in CD10H tumor cells than CD10L tumor cells

**Fig. 2:**
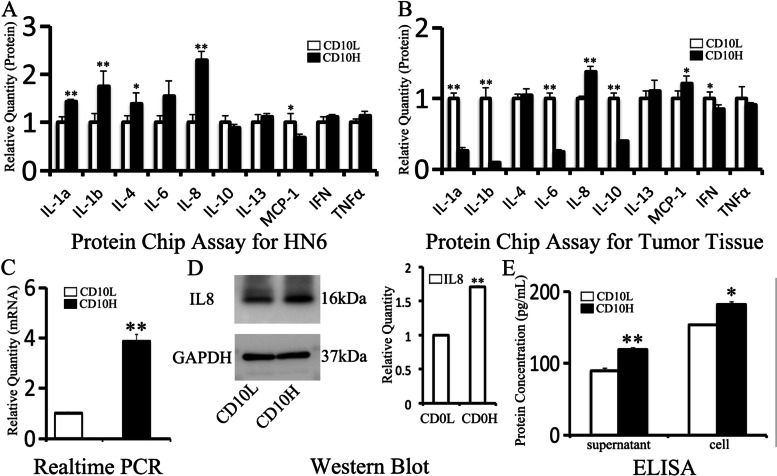
IL8 expression was higher in CD10H tumor cells. CD10H HN6 and CD10L HN6 cells were screened by MicroBead Kit. Protein chip assay was applied to screen the target gene in HN6 cells **(A)** and tumor tissue **(B)**. IL8 expression was verified by real-time PCR **(C)**, Western Blot **(D)**, and ELISA **(E)**. *P<0.05, **P<0.01

To explore the functional protein of CD10H tumor cells, the protein chip assay was applied to screen the target gene. In HN6 cells (2.3±0.18 v.s. 1±0.16, Fig. 2A) and OSCC tissue (1.37±0.09 v.s. 1±0.03, Fig. 2B), IL8 protein expression was higher in CD10H group compared to CD10L group; these data were verified by real-time PCR (3.87±0.28 v.s. 1.00±0.06, Fig. 2C), Western Blot (1.7±0.02 v.s. 1±0.01, Fig. 2D) and ELISA (119.54±0.47 v.s. 90.15±2.81 for supernatant; 181.86±3.91 v.s. 153.69±1.56 for cells, Fig. 2E).

### IL8 overexpression promoted CD10H HN6 migration, invasion, tumor formation, and spheroid formation

**Fig. 3:**
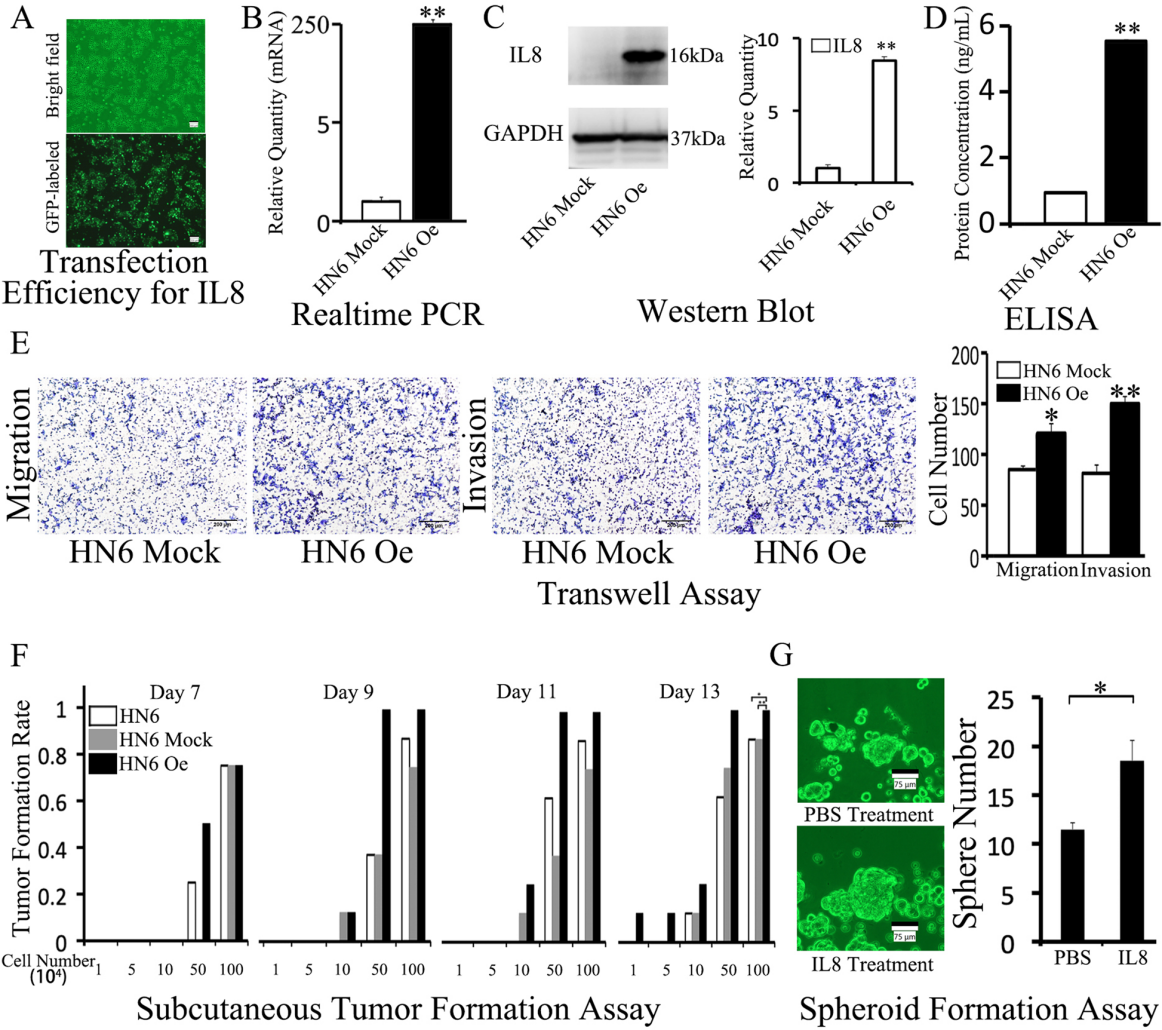
IL8 overexpression promoted HN6 migration, invasion, tumor formation, and spheroid formation**.** The transfection efficiency is shown in the microscope image **(A)**. IL8 expression was verified by real-time PCR **(B)**, Western Blot **(C)**, and ELISA **(D)**. Transwell assay was used to explore the migration and invasion ability between HN6 Oe and HN6 Mock **(E)**. Tumor formation was examined by xenograft animal model **(F)**. A spheroid formation assay was used to observe the spheroid formation when treated with IL8 **(G)**.

To evaluate the crucial role of IL8 involved in CD10H HN6, we established a high IL8 expression HN6 cells line (HN6 Oe) and its control HN6 cells (HN6 Mock). The transfection efficiency was higher than 90% (Fig. 3A); the IL8 expression level in the two cell lines was verified with real-time PCR (1±0.02 v.s. 239±0.19, Fig. 3B), Western Blot (1±0.24 v.s. 8.44±0.26, Fig. 3C), and ELISA (0.93±0.01 v.s. 5.53±0.05, Fig. 3D).

HN6 Oe cells showed higher migration and invasion ability than HN6 Mock cells (72±6 v.s. 121±10 for migration, 81±8 v.s. 150±7 for invasion, Fig. 3E). Moreover, the tumor formation rate was significantly higher in the HN6 Oe group than HN6 Mock and HN6 group at day 13 in the xenograft animal model (Fig. 3F). Also, IL8 promoted spheroid formation compared with the PBS group when IL8 cytokine was applied to the cell culture medium (12±1 v.s. 19±2, Fig. 3E).

### IL8 secretion in CD10H HN6 cells is regulated via the ERK signaling pathway

**Fig. 4:**
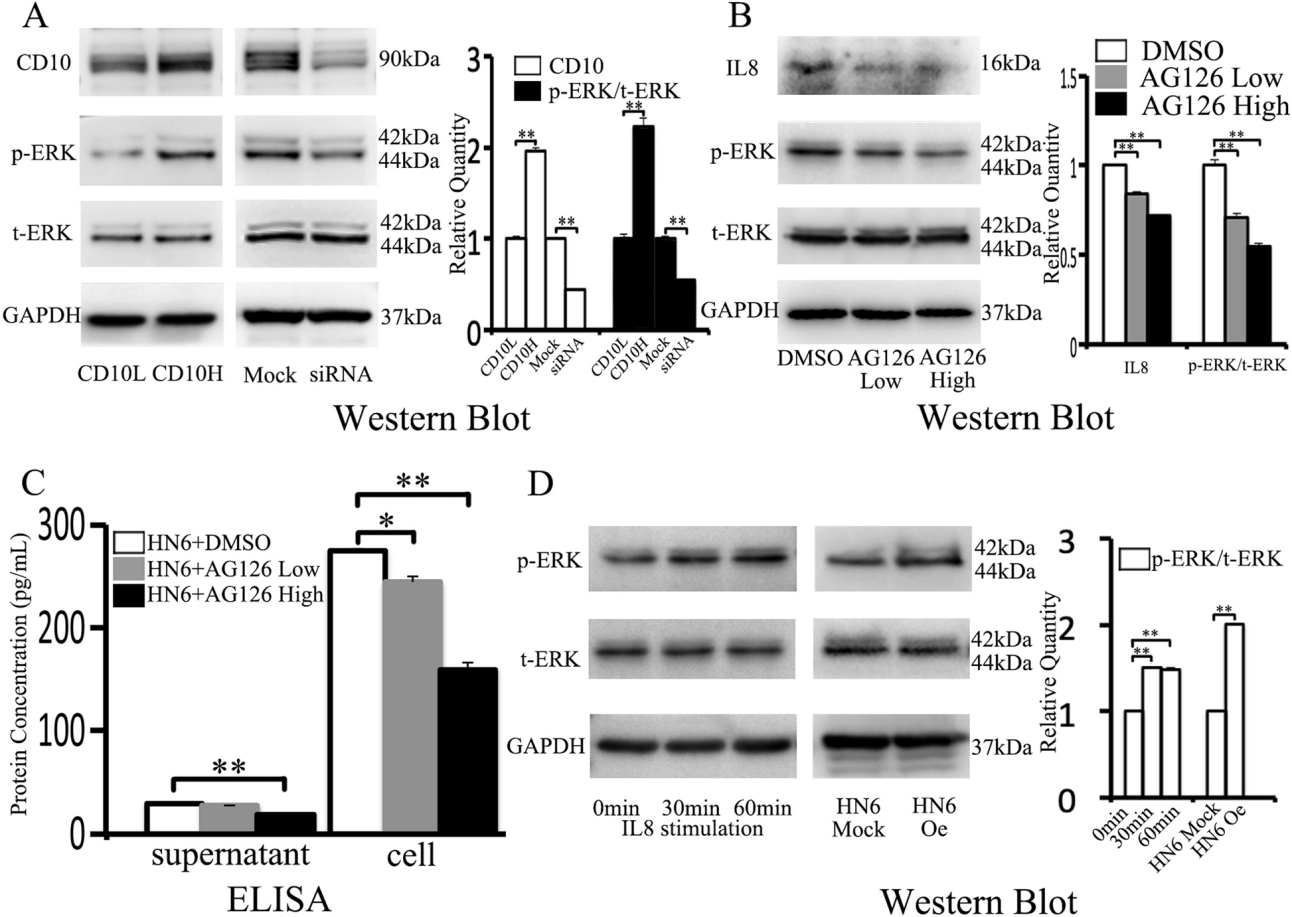
IL8 secretion in CD10H HN6 cells is regulated via the ERK signaling pathway. Expression of CD10, p-ERK, and t-ERK in CD10L, CD10H, Mock, and siRNA groups by Western blot **(A)**. Expression of CD10, p-ERK, and t-ERK in DMSO, AG126 Low, and AG126 High groups by Western blot **(B)**. IL8 expression in DMSO, AG126 Low, and AG126 High groups by ELISA **(C)**. p-ERK and t-ERK were evaluated by Western Blot when stimulated with IL8 and between HN6 Mock and HN6 Oe groups **(D)**.

ERK pathway can regulate the expression of IL8 in triple-negative breast cancer, thereby affecting the characteristics of breast cancer metastasis [27]. In this study, we analyzed whether the p-ERK signaling pathway is involved in the secretion of IL8 in CD10H HN6 cells. Western Blot showed that p-ERK expression was up-regulated in the CD10H group compared with the CD10L group (2.23±0.09 v.s. 1±0.05, Fig. 4A). The p-ERK expression was reduced when CD10 was down-regulated (1±0.03 v.s. 0.54±0.02, Fig. 4A). Furthermore, Western blot showed that IL8 expression was reduced in AG126 low and AG126 high groups when HN6 was treated with AG126, a specific p-ERK inhibitor (1±0.03 v.s. 0.71±0.02 v.s. 0.55±0.02, Fig. 4B). ELISA results (supernatant: 29.3±0 v.s. 27.6±0.7 v.s. 19.1±0.23; cell: 274.6±2.3 v.s. 243.7±7.0 v.s. 159.9±6.26, Fig. 4C) were consistent with Western Blot.

Next, we explored whether the increase of the IL8 factor could enhance the level of the ERK pathway in oral cancer cells. Western Blot showed that p-ERK expression was up-regulated with IL8 stimulation at 30min and 60min (1±0.01 v.s. 1.5±0.01 v.s. 1.49±0.01, Fig. 4D). The higher expression of p-ERK was also observed in HN6 Oe than in the HN6 Mock group (1±0.01 v.s. 2±0.02, Fig. 4D).

### IL8 secreted by CD10H HN6 cells promoted migration and invasion and restored tumor chemosensitivity

**Fig. 5:**
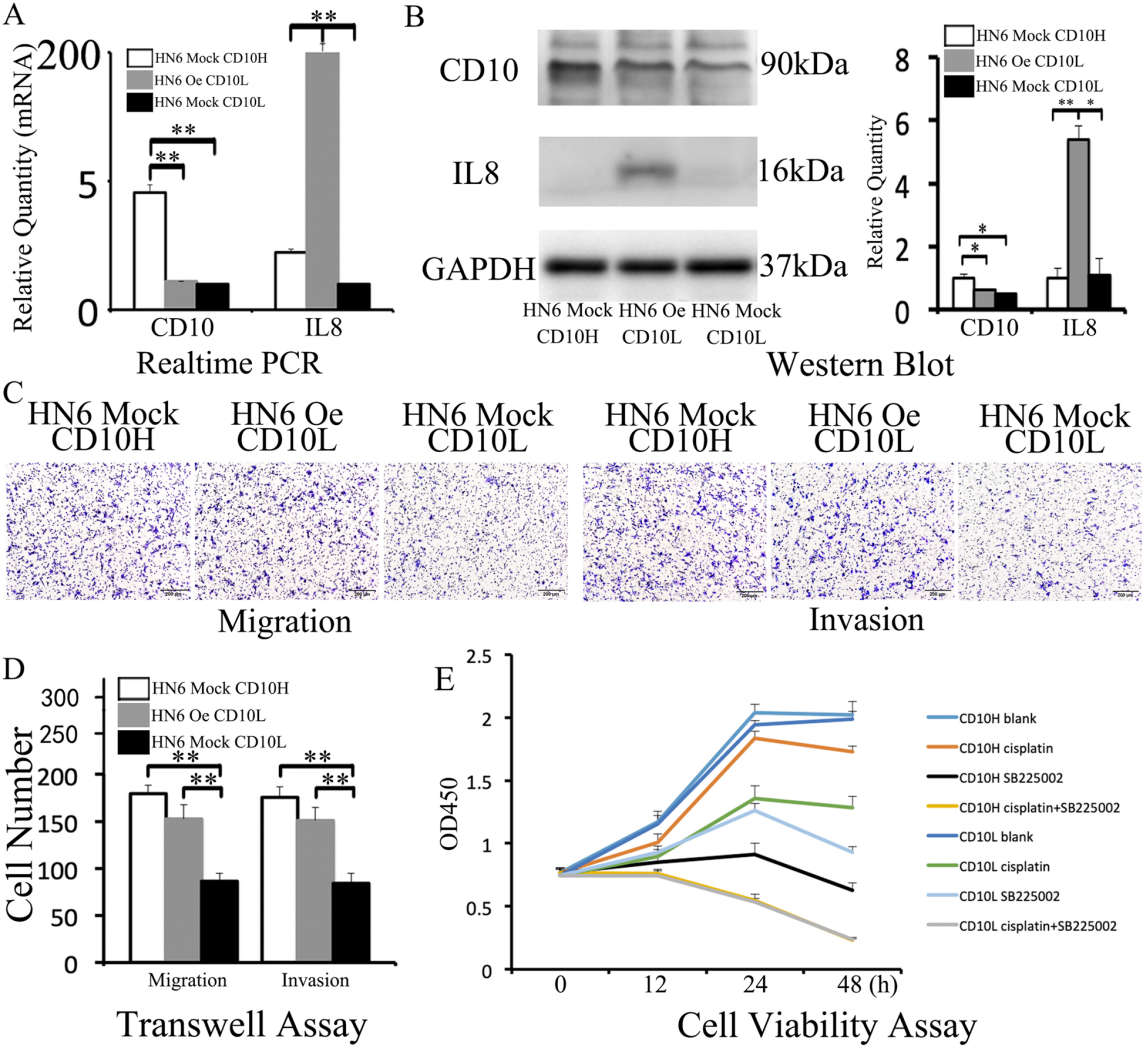
IL8 secreted by CD10H HN6 cells promoted migration and invasion and restores tumor chemosensitivity. HN6 Mock CD10H, HN6 Oe CD10L, and HN6 Mock CD10L were identified by real-time PCR **(A)** and Western blot **(B).** The three groups were compared by transwell assay to explore the ability of migration and invasion **(C&D)**. Cell viability of CD10H and CD10L HN6 cells were valued by CCK8 when treated with cisplatin and/or SB225002 **(E)**.

We screened HN6 Mock and HN6 Oe cells that expressed high and low levels of CD10. Real-time PCR (HN6 Mock CD10H v.s. HN6 Oe CD10L v.s. HN6 Mock CD10L: 4.54±0.31 v.s. 1.1±0.001 v.s. 1±0.003 for CD10, 2.23±0.13 v.s. 204.53±8.07 v.s. 1±0.004 for IL8, Fig. 5A) and Western Blot (HN6 Mock CD10H v.s. HN6 Oe CD10L v.s. HN6 Mock CD10L: 1±0.11 v.s. 0.63±0.01 v.s. 0.49±0.02 for CD10; 1±0.3 v.s. 5.4±0.44 v.s. 1.1±0.53 for IL8, Fig. 5B) were used to evaluate CD10 and IL8 expression. Next, we compared the migration (179±9 v.s. 153±15 v.s. 86±8, Fig. 5C&D) and invasion (176±11 v.s. 151±14 v.s. 84±11, Fig. 5C&D) ability in HN6 Mock CD10H, HN6 Oe CD10L, and HN6 Mock CD10L groups, respectively. Results showed that the migration and invasion were enhanced when IL8 was up-regulated in HN6 Oe CD10L than HN6 Mock CD10L.

Next, we examined the cell viability when cells were treated with cisplatin and/or SB225002 (IL8 specific inhibitor) (Fig. 5E). The cell viability of the CD10H group was higher than the CD10L group when treated with cisplatin; it was higher in the CD10L group compared to the CD10H group when treated with SB225002 at 24h and 48h. Both CD10H and CD10L had significantly lower cell viability than other groups when treated with SB225002 and cisplatin for 24h and 48h.

## Discussion

CD10 was reported for the first time in 2014 by Fukusumi *et al*, who discovered that CD10 is associated with therapeutic resistance and CSC-like properties of HNSCC. Since then, CD10 has been widely explored in the treatment of oral cancer. In our study, stemness-related markers BMI1, OCT4, and SOX2 were all up-regulated in CD10H HN6 cells. Overexpression of CD10 further promoted CSC-related genes expression and enhanced migration, invasion, spheroid formation, and chemoresistance in HN6 cells (Figure S1A-C). Meanwhile, CD10H HN6 cells exhibited more chemoresistance than CD10L HN6 cells.

Next, we further investigated the mechanism through which CD10H oral cancer cells regulate their biological behavior. We hypothesized that IL8 might regulate migration, invasion, and cisplatin resistance of CD10-positive oral cancer cells through the ERK pathway. Protein chip assay showed that IL8 was up-regulated in HN6 and CAL27 cells overexpressing CD10 (Figure S1D) as well as OSCC tissue. IL8, which is secreted by tumor cells [15, 28], tumor-associated fibroblast [16], and immune cells [29], participates in the development and progression of the tumor and has an important role in the tumor microenvironment. A phase II clinical trial found that overexpression of IL8 was significantly associated with shorter progression-free survival in patients with recurrent and/or metastatic squamous cell carcinoma of head and neck treated with dacomitinib [30]. Moreover, a recent clinical study showed that IL8 monoclonal antibody HuMax-IL8 is safe and well-tolerated in patients with metastatic or unresectable locally advanced solid tumors [13].

Next, we established the IL8 overexpression OSCC cell line HN6 Oe and controlled group HN6 Mock. IL8 overexpression promoted HN6 Oe migration, invasion, and spheroid formation *in vitro* and enhanced subcutaneous tumor formation *in vivo,* thus suggesting that IL8 has a functional role in HN6 cells.

Previous studies showed that IL8 expression is associated with p-ERK in breast cancer [31] and pancreatic adenocarcinoma [32]. Therefore, in this study, we investigated the relationship between p-ERK and IL8 expression. We found that the phosphorylation of p-ERK was up-regulated in CD10H HN6, while the knockdown of CD10 led to lower phosphorylation of p-ERK. In addition, the results showed that the phosphorylation level of p-ERK was reduced by AG126 as well as IL8 expression in a dose-dependent manner. These data suggested that CD10H HN6 cells expressed IL8 via the p-ERK signaling pathway. Meanwhile, IL8 could promote p-ERK phosphorylation in a time-dependent manner. Similar results were also seen in pancreatic cancer [33] and head and neck cancer [34]. This indicates that CD10H HN6 secrete IL8 via the phosphorylation of p-ERK, while IL8 promotes p-ERK phosphorylation. This mutual promotion created a microenvironment.

Additionally, we found that IL8 was expressed by CD10H tumor cells and created a tumor microenvironment to promote migration, invasion, and chemoresistance. HN6 Oe and HN6 Mock were screened by CD10 MicroBead Kit. We found that with the same level of CD10 expression in HN6 Oe CD10L and HN6 Mock CD10L groups, up-regulation of IL8 could promote the invasion and migration ability in HN6 cells. Meanwhile, CH10H HN6 was more sensitive to SB225002 and was more resistant to cisplatin than CD10L HN6. Applying SB225002 and cisplatin inhibited both CD10H and CD10L HN6 tumor cells. Also, IL8 promoted migration, invasion of tumor cells and was associated with CSCs and chemoresistance in many cancer types [35, 36]. Thus, we believe that targeting IL8 may increase the effectiveness of cisplatin treatment in OSCC.

## Conclusion

IL8 secretion by CD10 positive cells promotes migration, invasion, and cisplatin resistance to OSCC. This process is regulated by the p-ERK signaling pathway. Our results support the idea that the combined application of IL8 specific inhibitors and cisplatin enhances the treatment outcome in oral squamous cell carcinoma.

## Supplementary Information

**Additional file 1: Figure S1**. CD10 expression in CD10L CAL27 and CD10H CAL27 groups by real-time PCR **(A)** and Western blot **(B)**. Migration and invasion ability of CD10L CAL27 and CD10H CAL27 groups using Transwell assay **(C)**. The spheroid formation ability was evaluated by spheroid formation assay **(D)**. A protein chip assay was applied to screen the target gene in CAL27 cells **(E)**.

**Additional file 2: Figure S2**. Orginal gel images.

**Additional file 3.**

## Data Availability

The data that support the findings of this study are available from the corresponding author upon reasonable request.

## Abbreviations

CD10H: CD10 high
CD10L: CD10 low
CSC: Cancer stem cells
OSCC: Oral squamous cell carcinoma
