# Supplementary figures and images for: pERK-mediated IL8 secretion can enhance the migration, invasion, and cisplatin resistance of CD10-positive oral cancer cells
Source: BMC Cancer. 2021 Dec 1;21:1283. doi: 10.1186/s12885-021-09025-7 (PMC8638179; doi:10.1186/s12885-021-09025-7)

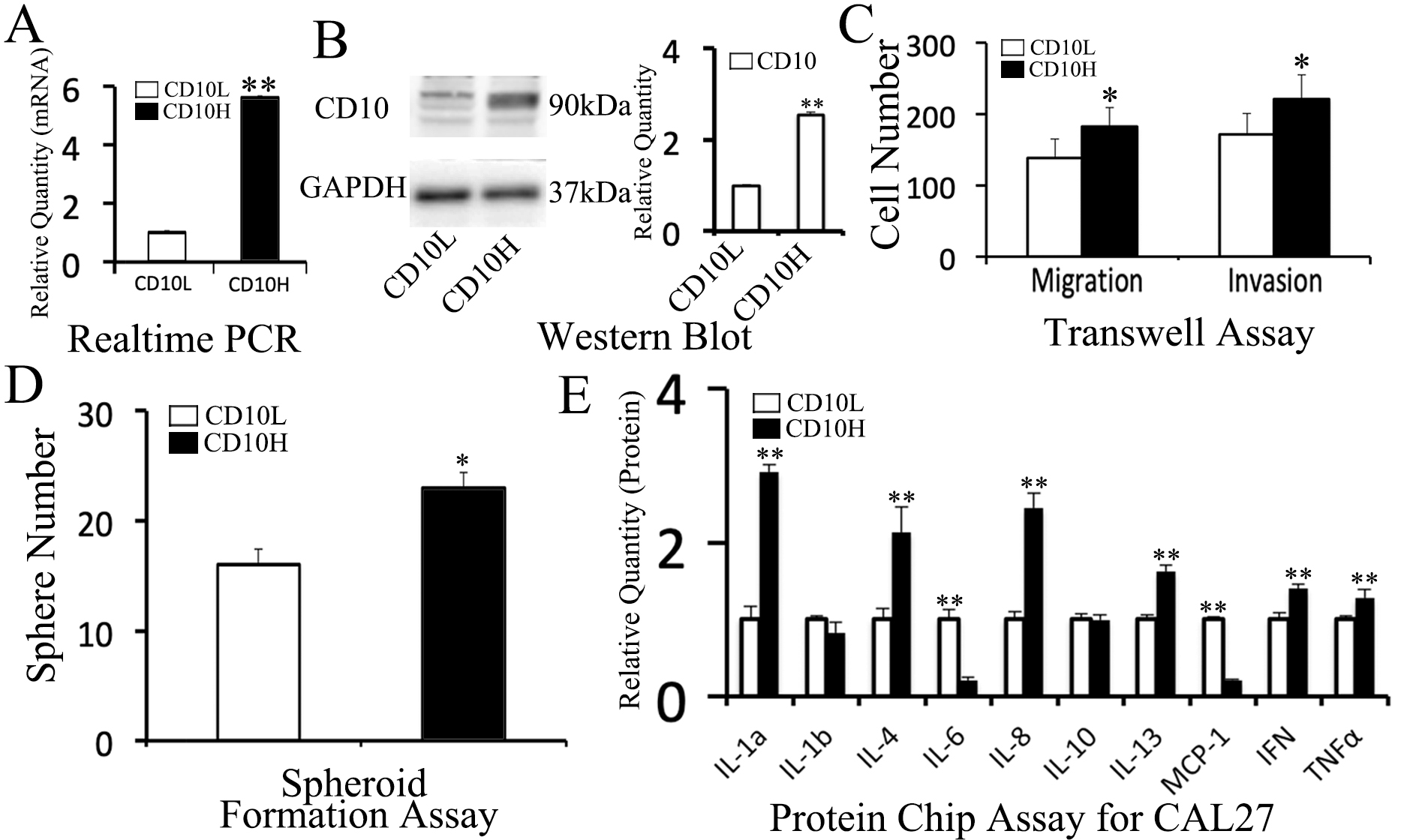

Supplement: Supplementary file 1 — Additional file 1: Figure S1. CD10 expression in CD10L CAL27 and CD10H CAL27 groups by real-time PCR (A) and Western blot (B). Migration and invasion ability of CD10L CAL27 and CD10H CAL27 groups using Transwell assay (C). The spheroid formation ability was evaluated by spheroid formation assay (D). A protein chip assay was applied to screen the target gene in CAL27 cells (E). [file 12885_2021_9025_MOESM1_ESM.jpg]

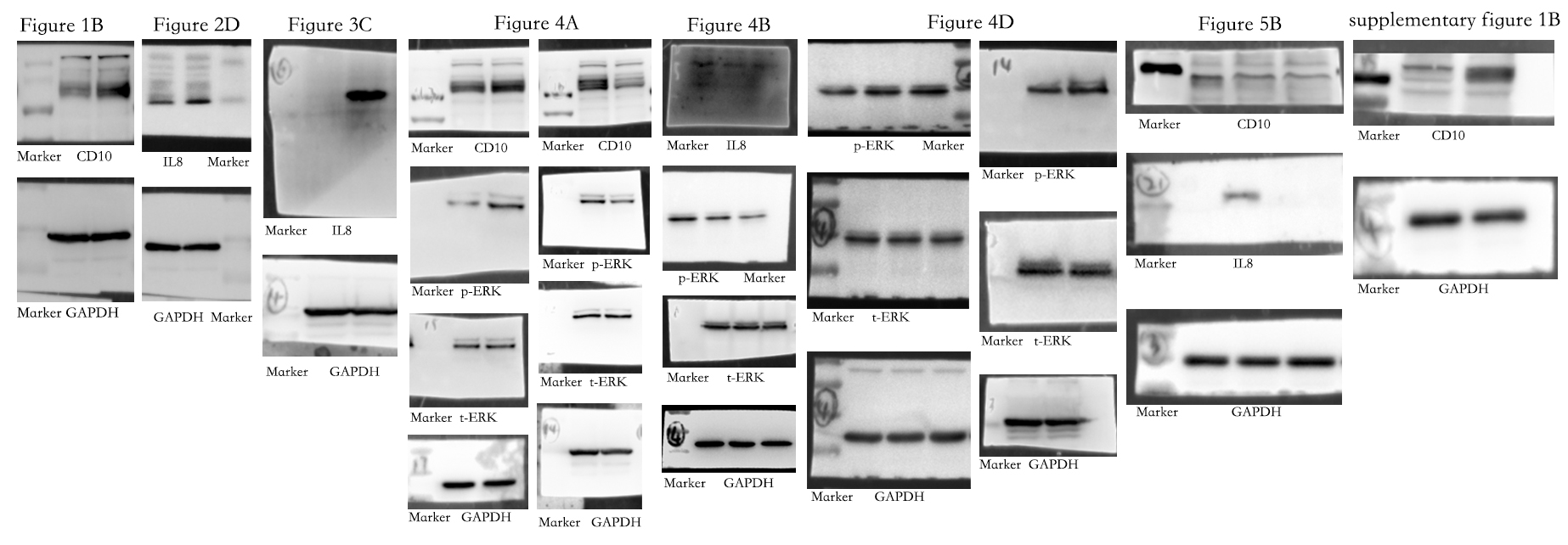

Supplement: Supplementary file 2 — Additional file 2: Figure S2. Orginal gel images. [file 12885_2021_9025_MOESM2_ESM.jpg]
