# Supplementary material for: pERK-mediated IL8 secretion can enhance the migration, invasion, and cisplatin resistance of CD10-positive oral cancer cells
Source: BMC Cancer. 2021 Dec 1;21:1283. doi: 10.1186/s12885-021-09025-7 (PMC8638179; doi:10.1186/s12885-021-09025-7)
Supplement: Supplementary file 3 — Additional file 3. [file 12885_2021_9025_MOESM3_ESM.docx]

Table S1

| Name | Gene ID | Primer sequences | |
| --- | --- | --- | --- |
| CD10 | 4311 | Forward | TGGATCTTGTAAGCAGCCTCA |
|  |  | Reverse | GCACAACGTCTCCAAGTTGC |
| BMI1 | 648 | Forward | CCACCTGATGTGTGTGCTTTG |
|  |  | Reverse | TTCAGTAGTGGTCTGGTCTTGT |
| OCT4 | 5460 | Forward | CTTGAATCCCGAATGGAAAGGG |
|  |  | Reverse | GTGTATATCCCAGGGTGATCCTC |
| SOX2 | 6657 | Forward | GCCGAGTGGAAACTTTTGTCG |
|  |  | Reverse | GGCAGCGTGTACTTATCCTTCT |
| IL8 | 3576 | Forward | CCACCGGAAGGAACCATCTC |
|  |  | Reverse | TTCCTTGGGGTCCAGACAGA |
